# Whole brain and deep gray matter atrophy detection over 5 years with 3T MRI in multiple sclerosis using a variety of automated segmentation pipelines

**DOI:** 10.1371/journal.pone.0206939

**Published:** 2018-11-08

**Authors:** Renxin Chu, Gloria Kim, Shahamat Tauhid, Fariha Khalid, Brian C. Healy, Rohit Bakshi

**Affiliations:** 1 Department of Neurology, Brigham and Women's Hospital, Harvard Medical School, Boston, Massachusetts, United States of America; 2 Departments of Radiology, Brigham and Women's Hospital, Harvard Medical School, Boston, Massachusetts, United States of America; Iwate Medical University, JAPAN

## Abstract

**Background:**

Cerebral atrophy is common in multiple sclerosis (MS) and selectively involves gray matter (GM). Several fully automated methods are available to measure whole brain and regional deep GM (DGM) atrophy from MRI.

**Objective:**

To assess the sensitivity of fully automated MRI segmentation pipelines in detecting brain atrophy in patients with relapsing-remitting (RR) MS and normal controls (NC) over five years.

**Methods:**

Consistent 3D T1-weighted sequences were performed on a 3T GE unit in 16 mildly disabled patients with RRMS and 16 age-matched NC at baseline and five years. All patients received disease-modifying immunotherapy on-study. Images were applied to two pipelines to assess whole brain atrophy [brain parenchymal fraction (BPF) from SPM12; percentage brain volume change (PBVC) from SIENA] and two other pipelines (FSL-FIRST; FreeSurfer) to assess DGM atrophy (thalamus, caudate, globus pallidus, putamen). MRI change was compared by two sample t-tests. Expanded Disability Status Scale (EDSS) and timed 25-foot walk (T25FW) change was compared by repeated measures proportional odds models.

**Results:**

Using FreeSurfer, the MS group had a ~10-fold acceleration in on-study volume loss than NC in the caudate (mean decrease 0.51 vs. 0.05 ml, p = 0.022). In contrast, caudate atrophy was not detected by FSL-FIRST (mean decrease 0.21 vs. 0.12 ml, p = 0.53). None of the other pipelines showed any difference in volume loss between groups, for whole brain or regional DGM atrophy (all p>0.38). The MS group showed on-study stability on EDSS (p = 0.47) but slight worsening of T25FW (p = 0.054).

**Conclusions:**

In this real-world cohort of mildly disabled treated patients with RRMS, we identified ongoing atrophy of the caudate nucleus over five years, despite the lack of any significant whole brain atrophy, compared to healthy controls. The detectability of caudate atrophy was dependent on the MRI segmentation pipeline employed. These findings underscore the increased sensitivity gained when assessing DGM atrophy in monitoring MS.

## Introduction

Brain atrophy is common, progressive, and begins early in the disease course of multiple sclerosis (MS). Numerous studies have shown the high clinical relevance of brain atrophy in predicting physical disability and cognitive impairment in patients with MS [1, 2]. Furthermore, this brain atrophy is only partly related to conventional MS-related white matter (WM) lesions and thus the measurement of atrophy provides unique information about the destructive aspects of the disease [3]. Global and compartment-specific or regional atrophy can be estimated from MRI scans. The most commonly-assessed aspect of brain atrophy is whole brain volume, due to the availability of numerous highly reliable and sensitive methods for its measurement [1, 4]. Analysis of regional brain volume also has important implications related to clinical impairment, disease progression, and therapeutic monitoring [5, 6]. Gray matter (GM) tissue loss is of particular importance because of its functional relevance. Several studies have shown that damage to this tissue is more clinically relevant than WM volume loss or lesion changes in MS [7–9].

Cerebral GM is classed as either cortical or deep gray matter (DGM). While both of these areas of GM are typically affected in MS, DGM is an early and selectively affected site [10, 11]. Histologic analysis has shown at least two processes occurring in the DGM, both demyelinating foci and widespread tissue degeneration [12]. Demyelination is shown to be present in the early stages of the disease, and both processes are associated with oxidative injury [12]. Neurodegeneration is associated with reduced neuronal density, oligodendrocyte and axonal injury, lymphocyte infiltration, microglial activation, and iron deposition [12]. Furthermore, DGM injury clearly has clinical relevance in patients with MS, as several studies have shown [13–16]. Among the many potential uses of measuring DGM damage in people with MS, the longitudinal assessment of atrophy in these structures by automated segmentation from MRI may provide an efficient, sensitive, and reliable tool to assess neurotherapeutic effects [6, 17].

Currently, global and regional brain atrophy can be assessed using a wide variety of MRI post-processing algorithms [1–8, 11, 14, 16–19]. Automated or semi-automated measurement techniques fall mainly into two categories: registration- and segmentation-based [20]. Registration-based methods measure within-subject change in brain volume between scans on a voxel-by-voxel basis, to identify shifts in brain structure [21]. Segmentation techniques using static comparisons of volumetric data between two scans of the same subject, with each scan usually normalized to the subject’s intracranial cavity; such normalization may be residual [atlas-based: e.g. normalized brain parenchymal volume (BPV)] [20, 22] or proportional [scaled to the patient’s own intracranial cavity; e.g. brain parenchymal fraction (BPF)] [20, 23]. The measurement of regional brain atrophy also includes a variety of approaches/pipelines [24–26]. Studies have shown that the results from different pipelines to measure brain atrophy are not necessarily interchangeable [20, 26, 27] and may lead to divergent conclusions regarding MS therapeutic efficacy [28, 29].

The objective of our study was to assess the sensitivity of a range of fully automated MRI segmentation pipelines in assessing whole brain and regional DGM volume and their atrophy over five years in patients with relapsing-remitting multiple sclerosis (RRMS) and normal controls (NC) from high-resolution 3T MRI scans.

## Methods

### Ethics statement

All participants provided written informed consent to participate in the study. This consent procedure was approved by our ethics committee. The Partners Human Research Committee approved this study.

### Subjects and neurologic examination

Demographic and clinical characteristics are summarized in Table 1 and in the supporting information (S1 File). Sixteen patients with MS and 16 NC underwent baseline and 5-year follow-up scans. MS patients met the International Panel criteria for either relapsing MS or a clinically isolated syndrome (CIS) [30]. All patients underwent an examination by MS specialist neurologist including evaluation of the Expanded Disability Status Scale (EDSS) [31] score and timed 25-foot walk (T25FW) [32]. All patients received disease-modifying immunotherapy during the observation period, as was selected and prescribed by their treating physician according to routine clinical care.

**Table 1:** Demographic and clinical characteristics.

	Multiple sclerosis	Normal controls	p-value^
Number of subjects	16	16	
Sex ratio (women/men)	0.69 (11/5)	0.63 (10/6)	0.71
Age at baseline (years)	45.1±8.4 (29.6–57.2)	42.6±8.7 (23.1–58.7)	0.42
Time from baseline to follow-up MRI, months	56.7±6.6 (49.0–71.0)	56.8±6.5 (48.0–66.0)	0.98
Multiple sclerosis disease category	relapsing-remitting	-	
Disease duration on baseline scan, years^†^	13.4±10.5 (1.3–31.7)	-	
Disease duration on follow-up scan, years^†^	18.2±10.2 (6.7–36.5)	-	
EDSS score (baseline)	1.3±1.0 (0–3.5)	-	
EDSS score (follow-up)	1.8±1.8 (0–6.0)	-	
Timed 25-foot walk (baseline), seconds	4.4±0.6 (3.5–5.2)	-	
Timed 25-foot walk (follow-up), seconds	4.9±0.8 (4.0–6.1)	-	

Data are shown as mean±standard deviation (range) unless otherwise indicated; EDSS = Expanded Disability Status Scale

^†^Time from first symptom.

^p values are from comparisons between the multiple sclerosis and normal control groups–see Methods section for statistical methods descriptions.

### MRI acquisition

All subjects underwent brain MRI at 3T (Signa Excite; GE Healthcare). A consistent coronal 3D T1-weighted modified driven equilibrium Fourier transform (MDEFT) pulse sequence was performed (TR: 7.9 ms, TE: 3.14 ms, flip angle: 15°, number of slices: 124, FOV: 24×24 cm, voxel size: 0.94×0.94×1.6 mm3). The total scan time was 7.5 minutes.

### Reproducibility experiment and scanner upgrade

During the study, by decision of the hospital, the scanner underwent a software and hardware upgrade (gradient coil amplifiers, RF receiver system and software). This was out of our control as there was no intention to do this study related to a scanner upgrade. To investigate scanner effects before and after the upgrade, we also performed a reliability study. Eleven subjects (4 MS and 7 NC) underwent scan-rescan pairs with an average of 7 days between scans (range 0 to 42 days), without an intervening upgrade. In addition, to assess the effect of the upgrade, 3 subjects (2 MS and 1 NC) also underwent a scan-rescan before and after the scanner upgrade scan with an average of 51 days between scans (range 34 to 78 days).

### MRI analysis

**Fig 1:**
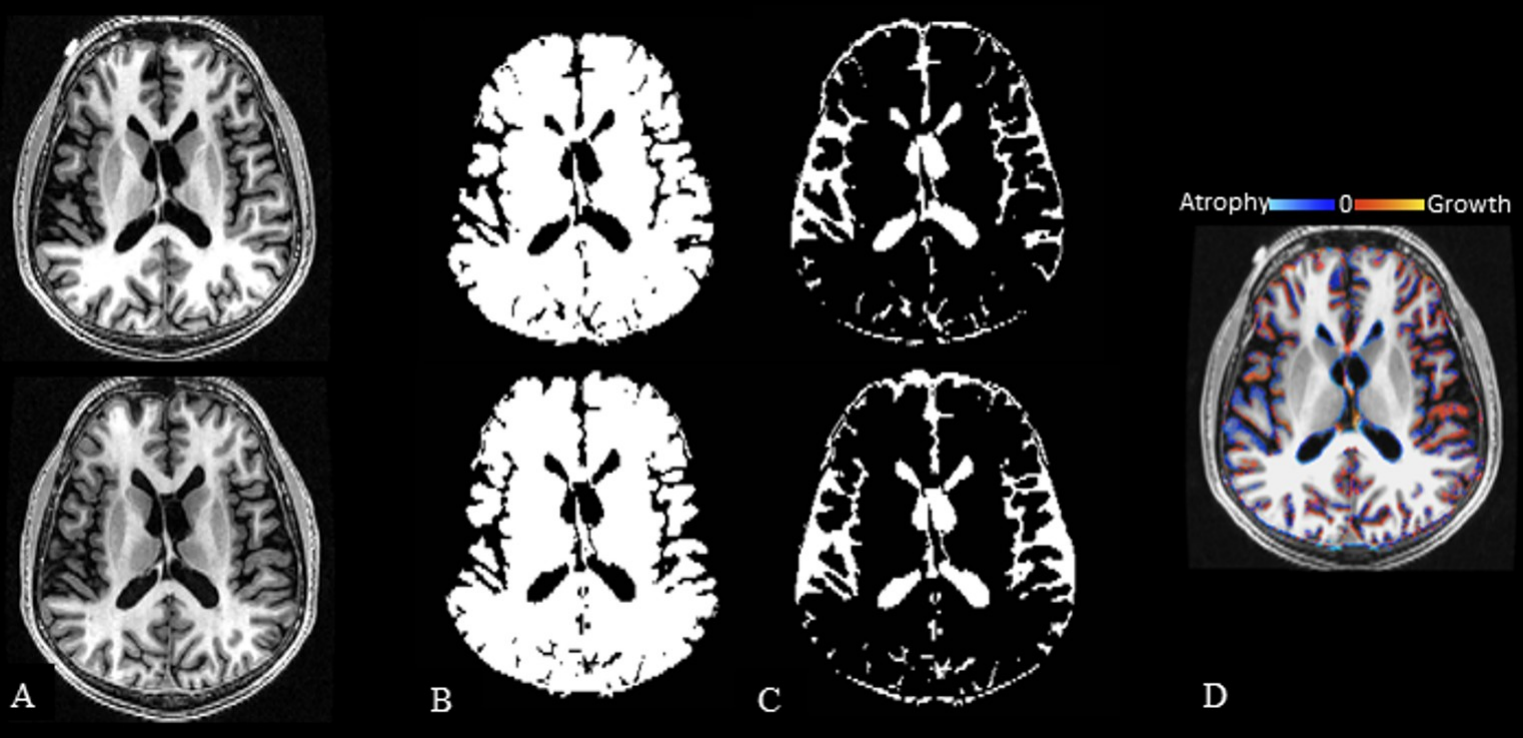
Two fully automated segmentation pipelines used to determine change in whole brain volume. 3T T1-weighted modified driven equilibrium Fourier transform pulse sequence–reformatted axial images. Panels A-C show baseline images in the top row and follow-up images in the bottom row. A: source images; B/C: SPM12 tissue class segmentation maps (brain parenchyma–B, CSF–C), used to calculate brain parenchymal fraction (BPF). Panel D shows a sample SIENA percentage brain volume change (PBVC) map comparing baseline to follow-up images from this anatomic section. Images are from a 51-year-old man with relapsing-remitting multiple sclerosis at baseline and 4.5 years later; baseline disease duration = 30.1 years; baseline/follow-up Expanded Disability Status Scale score = 0/0, timed 25-foot walk = 5.0/4.0 seconds, and BPF = 0.802/0.798. PBVC was -0.9% (decreased) over the study period. SPM12 = statistical parametric mapping, v. 12; SIENA = structural image evaluation, using normalization, of atrophy, v. 5.0 (see Methods section for details).

**Fig 2:**
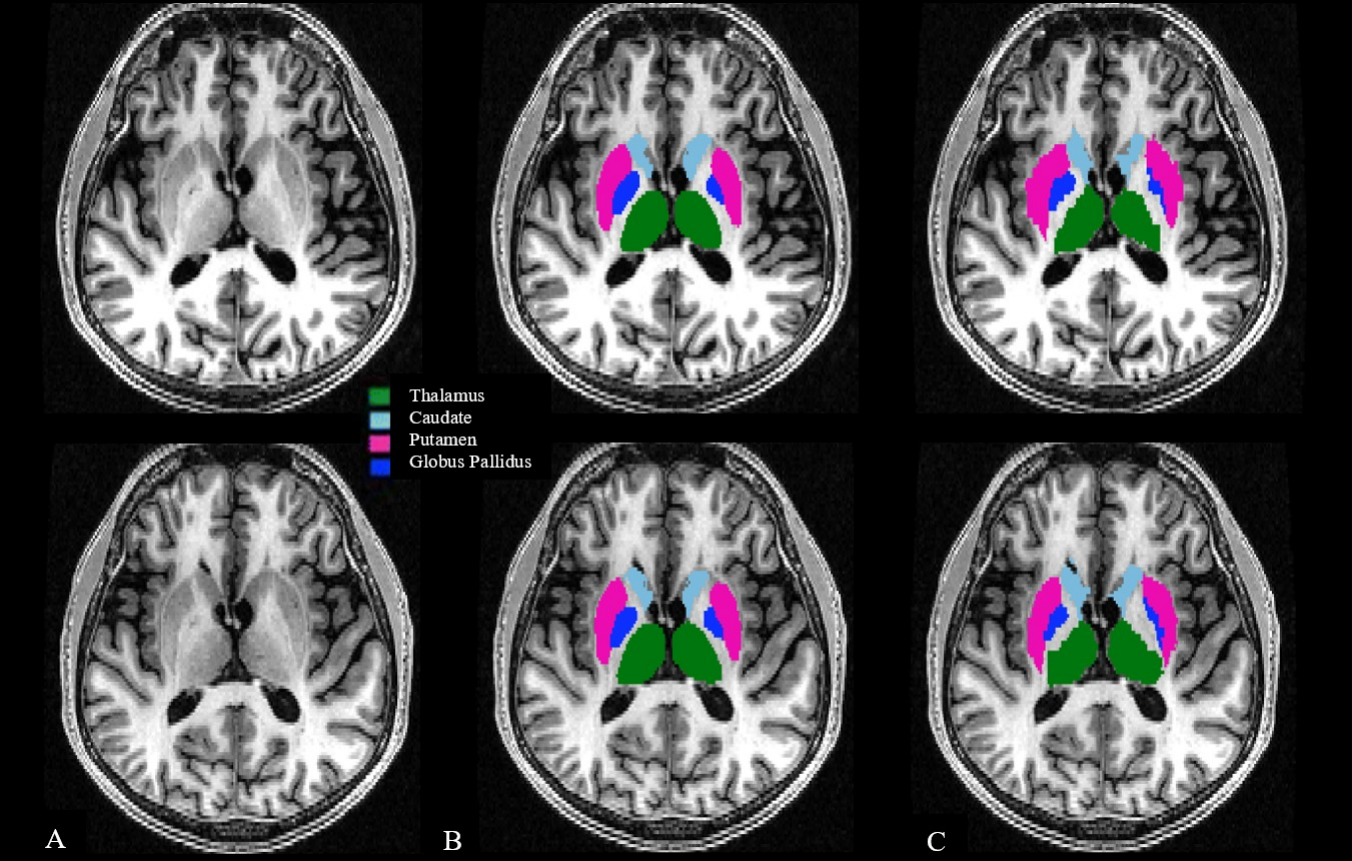
Segmentation of cerebral subcortical deep gray matter with FSL-FIRST and FreeSurfer pipelines. 3T T1-weighted modified driven equilibrium Fourier transform pulse sequence axial re-sampled images. Panels A-C show baseline images in the top row and follow-up images in the bottom row. A: source images; B/C: Subcortical deep gray matter (DGM) segmentation; B: FSL-FIRST segmentation (v. 5.0); C: FreeSurfer segmentation (v. 5.3.0). Segmentation maps are overlaid to raw re-sampled 3D T1-weighted images. Total DGM volume (baseline/follow-up) was: 40.92/39.66 ml for FSL-FIRST and 41.78/41.12 ml for FreeSurfer. Images are over 4.5 years from a man with relapsing-remitting multiple sclerosis. At baseline/follow-up, status was: age = 51.7/56.1 years, disease duration = 30.1/34.5 years, Expanded Disability Status Scale score = 0/0, timed 25-foot walk = 5.0/4.0 seconds. Total DGM = thalamus + caudate + putamen + globus pallidus.

All original DICOM images were converted to NIfTI format using Jim (v. 7.0, http://www.xinapse.com/) and were maintained in their native coronal slice orientation. Images were applied to two fully automated pipelines to assess normalized whole brain volume change [brain parenchymal fraction (BPF) from SPM12, http://www.fil.ion.ucl.ac.uk/spm/software/spm12; percentage brain volume change (PBVC) from SIENA (v.5.0; https://fsl.fmrib.ox.ac.uk)] (Fig 1). In addition, two fully automated pipelines FSL-FIRST (v.5.0, https://fsl.fmrib.ox.ac.uk) and FreeSurfer (v.5.3.0, https://surfer.nmr.mgh.harvard.edu) assessed the volume of the DGM (thalamus, caudate, globus pallidus, putamen, Fig 2). The supporting information (S1 File), provided with this manuscript, is a spreadsheet that includes all subjects’ volumetric/segmentation results calculated from all four pipelines.

### Statistical analysis

All statistical analysis was completed with the statistical package Stata/IC (v. 14.2, StataCorp LLC, College Station, TX). The demographic characteristics (Table 1) of the MS patients and normal controls were compared using a chi-squared test for categorical variables and a t-test for continuous variables. A paired t-test was used to estimate the mean within person change (baseline vs. follow-up) in each of the MRI measures in the normal controls and MS patients separately. The difference in the mean within person change was compared between the two groups using a two sample t-test. The change in the EDSS and T25FW was assessed by repeated measures proportional odds models with a patient specific random effect to account for the within patient correlation.

## Results

### Whole brain volume change: Patients vs. controls

**Fig 3:**
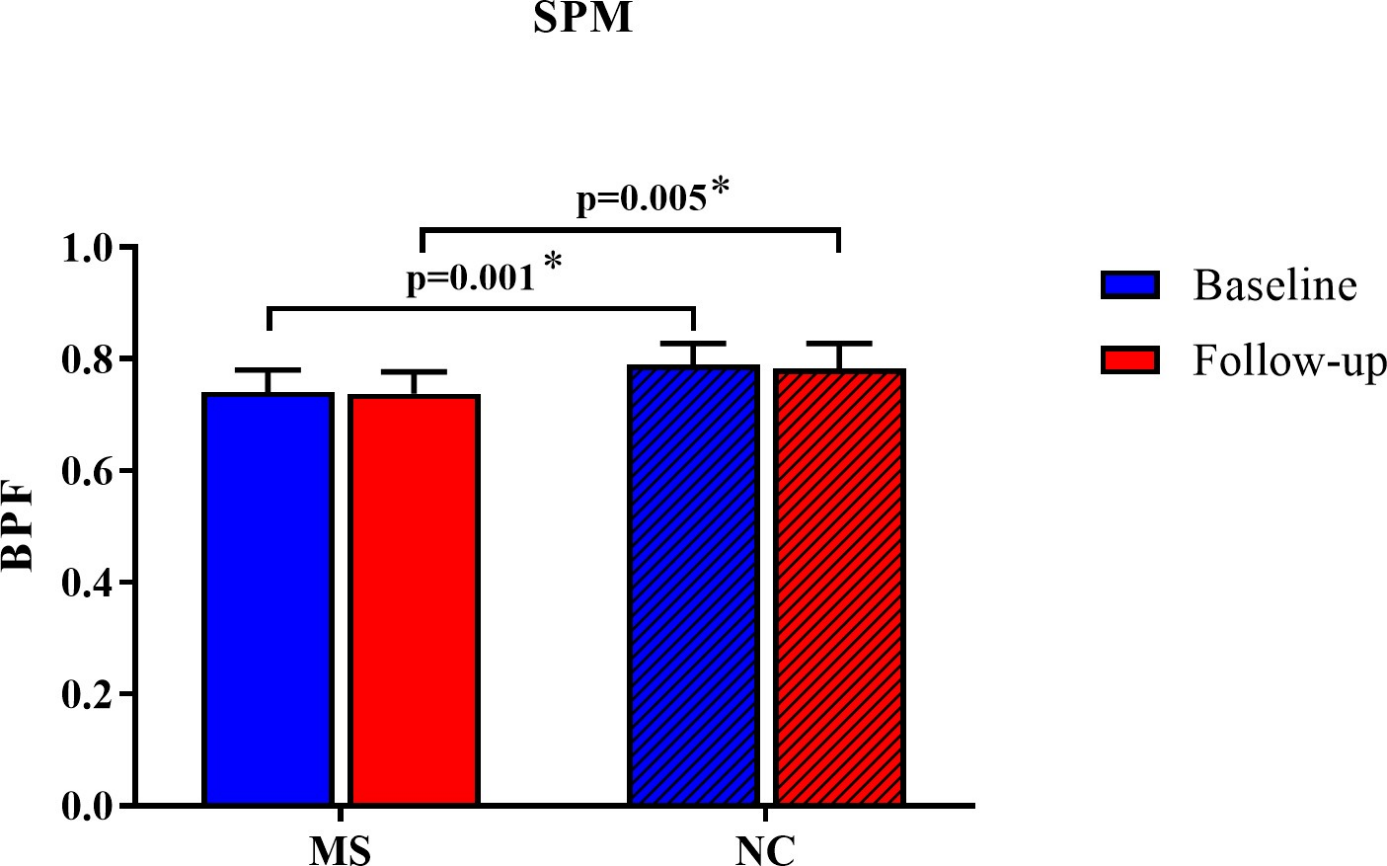
Normalized whole brain volume change over 5 years using an SPM pipeline. At both baseline and follow-up time points, the MS group had lower BPF than the NC group (both p<0.01). However, there was no significant BPF change during the observation period in either group (both p>0.3, Table 2). Furthermore, there was no significant difference in the on-study absolute or percent change between the MS and NC groups (both p>0.6, Table 2). Data are shown as means with standard deviations. BPF = brain parenchymal fraction; MS = multiple sclerosis; NC = normal controls; SPM = statistical parametric mapping, v. 12; *p<0.05.

**Fig 4:**
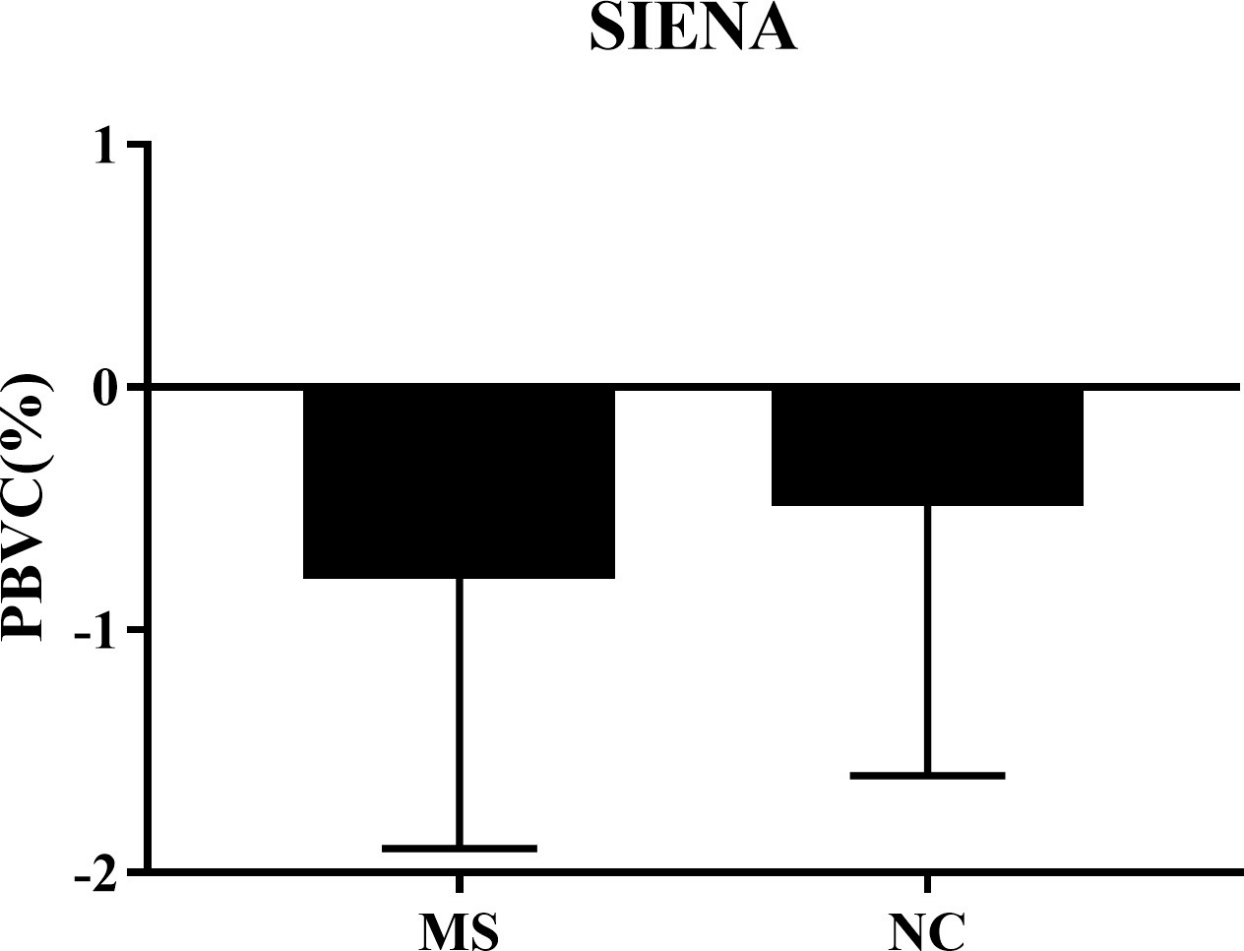
Whole brain volume change over 5 years using the SIENA pipeline. Comparing baseline and follow-up time point images using SIENA, there was no significant difference in the on-study PBVC between the MS and NC groups (p = 0.44, Table 2). Data are shown as means with standard deviations. MS = multiple sclerosis; NC = normal controls; PBVC = percentage brain volume change between baseline and follow-up (a negative number indicates brain volume loss over time); SIENA = structural image evaluation, using normalization, of atrophy, v. 5.0.

As shown in Table 2, Fig 3 and Fig 4, at both baseline and follow-up time points, the MS group had lower BPF than the NC group, as detected by the SPM12 segmentation pipeline (both p<0.01). However, the SPM12 pipeline did not show any significant BPF change during the observation period in either group (both p>0.3, Table 2). Furthermore, the SPM12 and SIENA pipelines did not show any significant difference in the on-study change in whole brain volume between the MS and NC groups (both p>0.4, Table 2). Thus, neither analysis pipeline was able to demonstrate significant whole brain atrophy in the MS group as compared to the NC group.

**Table 2:** Whole brain volume change over 5 years.

Pipeline		Multiple sclerosis	Normal controls	p-value (between groups)
SPM	Baseline BPF	0.741 (0.039)	0.790 (0.038)	0.001*
	Follow-up BPF	0.738 (0.039)	0.783 (0.045)	0.005*
	Change in BPF (p-value within group)	-0.003 (0.023) p = 0.63	-0.007 (0.026) p = 0.32	0.67
	Percent change in BPF	-0.33 (3.21)	-0.81 (3.24)	0.68
SIENA	PBVC	-0.79 (1.11)	-0.49 (1.11)	0.44

Data are shown as mean (standard deviation); BPF = brain parenchymal fraction; PBVC = percentage brain volume change between baseline and follow-up (a negative number indicates brain volume loss over time); SPM = statistical parametric mapping, v. 12; SIENA = structural image evaluation, using normalization, of atrophy, v. 5.0

*p<0.05

### Deep gray matter volume change: Patients vs. controls

**Fig 5:**
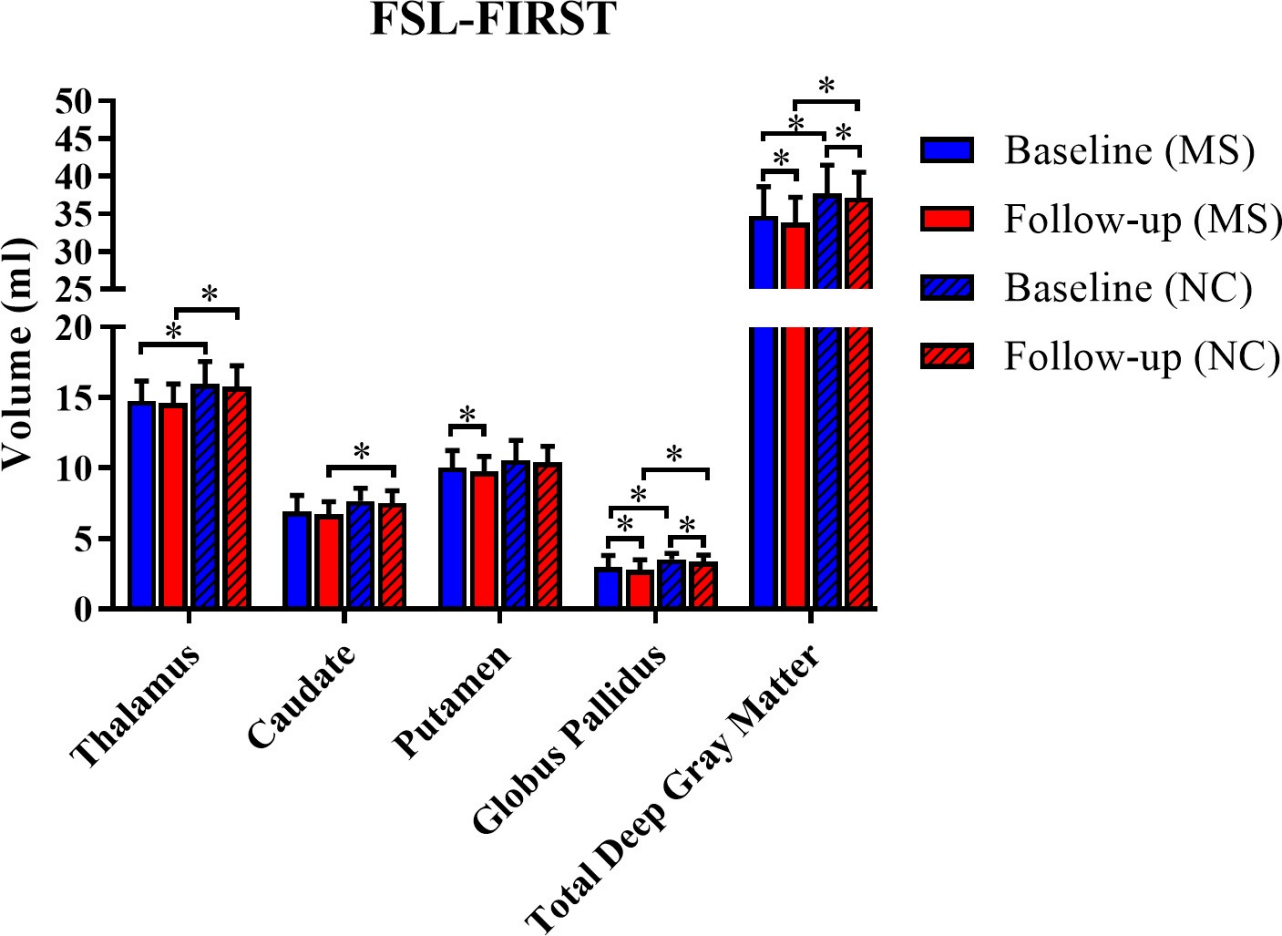
Cerebral deep gray matter volume change over 5 years with the FSL-FIRST pipeline. Individual nuclei and total DGM volume measurements are shown for both baseline and follow-up. Several of the volumes were significantly lower in the MS vs. NC groups at one or both time points (Table 3). With regard to within group on-study change, significant atrophy was detected during the 5-year period in the putamen (MS group), globus pallidus (both groups), and total DGM (both groups) (all p<0.05, Table 3). However, when examining between group (MS vs. NC) on-study change between baseline and follow-up observations, no significant differences were noted (all p>0.05). Data are shown as means with standard deviations. DGM = cerebral subcortical deep gray matter; MS = multiple sclerosis; NC = normal controls; FSL-FIRST = FMRIB’s integrated registration & segmentation tool, v. 5.0; total DGM = cerebral subcortical deep gray matter = thalamus + caudate + putamen + globus pallidus; *p<0.05.

**Fig 6:**
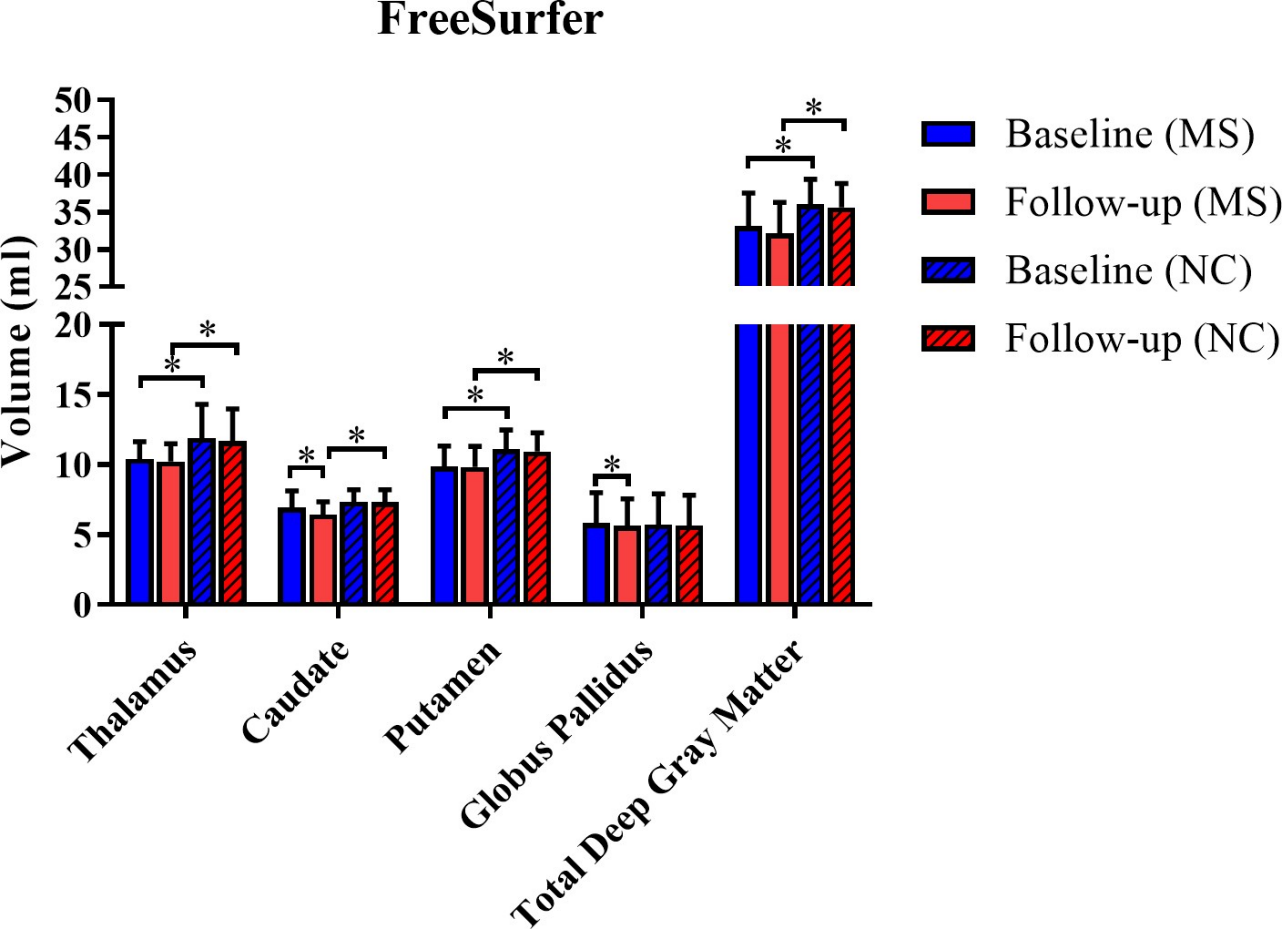
Cerebral deep gray matter volume change over 5 years with the FreeSurfer pipeline. Individual nuclei and total DGM volume measurements are shown for both baseline and follow-up. Several of the volumes were significantly lower in the MS vs. NC groups at one or both time points (Table 3). Regarding within group on-study change, only the caudate and globus pallidus in the MS group showed significant atrophy during the 5-year period (both p<0.05, Table 3). Furthermore, when examining between group (MS vs. NC) on-study change between baseline and follow-up observations, the rate of caudate volume loss was lower in the MS vs. NC group (p<0.05, Table 3). Data are shown as means with standard deviations. DGM = cerebral subcortical deep gray matter; MS = multiple sclerosis; NC = normal controls; total DGM = cerebral subcortical deep gray matter = thalamus + caudate + putamen + globus pallidus; *p<0.05.

As shown in Table 3, Fig 5 and Fig 6, with regard to within group on-study change, significant atrophy was detected by the FSL-FIRST segmentation pipeline during the 5-year period in the putamen (MS group), globus pallidus (both groups), and total DGM (both groups) (all p<0.05). In addition, significant atrophy was detected by the FreeSurfer segmentation pipeline during the 5 year period in the caudate (MS group) and globus pallidus (MS group) (both p<0.05, Table 3). When comparing the on-study difference between baseline and follow-up between the MS and NC groups, the MS group had a ~10-fold acceleration in on-study volume loss in the caudate volume detected by the FreeSurfer pipeline (mean decrease 0.51 vs. 0.05 ml, p = 0.022). Thus, both software analysis pipelines were able to demonstrate significant regional DGM atrophy in both groups. However, the topography of atrophy detection differed between pipelines. Furthermore, only one of the pipelines showed a higher on-study rate of atrophy in the MS group as compared to the NC group.

**Table 3:** Cerebral deep gray matter volume change over 5 years.

Structure		FSL-FIRST			FreeSurfer		
		MS	NC	p-value MS vs. NC	MS	NC	p-value MS vs. NC
Thalamus	Baseline	14.77 (1.41)	15.96 (1.60)	0.033*	10.42 (1.21)	11.90 (2.41)	0.036*
	Follow-up	14.62 (1.34)	15.78 (1.47)	0.027*	10.24 (1.26)	11.69 (2.28)	0.033*
	Change	-0.15 (0.73) p = 0.41	-0.18 (0.37) p = 0.066	0.89	-0.19 (0.65) p = 0.27	-0.21 (0.56) p = 0.15	0.90
	Percent change	-0.87 (5.54) p = 0.54	-1.06 (2.26) p = 0.080	0.90	-1.61 (7.24) p = 0.39	-1.56 (4.09) p = 0.15	0.98
Caudate	Baseline	6.92 (1.14)	7.66 (0.90)	0.051	6.96 (1.15)	7.34 (0.88)	0.30
	Follow-up	6.71 (0.90)	7.53 (0.85)	0.012*	6.45 (0.90)	7.30 (0.91)	0.013*
	Change	-0.21 (0.50) p = 0.11	-0.12 (0.27) p = 0.091	0.53	-0.51 (0.72) p = 0.012*	-0.05 (0.28) p = 0.52	0.022*
	Percent change	-2.45 (7.43) p = 0.21	-1.50 (3.35) p = 0.094	0.64	-6.55 (9.05) p = 0.011*	-0.63 (3.94) p = 0.53	0.023*
Putamen	Baseline	10.04 (1.21)	10.57 (1.39)	0.26	9.86 (1.47)	11.11 (1.37)	0.019*
	Follow-up	9.74 (1.10)	10.40 (1.14)	0.10	9.82 (1.50)	10.94 (1.34)	0.034*
	Change	-0.30 (0.44) p = 0.014*	-0.17 (0.54) p = 0.23	0.46	-0.03 (0.87) p = 0.88	-0.16 (0.53) p = 0.23	0.61
	Percent change	-2.84 (4.22) p = 0.017*	-1.28 (5.14) p = 0.33	0.36	-0.00 (9.01) p = 0.99	-1.37 (4.86) p = 0.28	0.60
Globus pallidus	Baseline	2.98 (0.81)	3.52 (0.42)	0.023*	5.84 (2.15)	5.72 (2.20)	0.87
	Follow-up	2.78 (0.73)	3.39 (0.44)	0.007*	5.63 (1.92)	5.66 (2.16)	0.96
	Change	-0.20 (0.27) p = 0.010*	-0.13 (0.19) p = 0.015*	0.41	-0.21 (0.37) p = 0.036*	-0.05 (0.38) p = 0.060	0.24
	Percent change	-5.86 (9.50) p = 0.026*	-3.65 (5.16) p = 0.013*	0.42	-2.13 (6.95) p = 0.24	-0.57 (7.24) p = 0.76	0.54
Total DGM	Baseline	34.71 (3.87)	37.71 (3.76)	0.033*	33.09 (4.41)	36.07 (3.30)	0.039*
	Follow-up	33.84 (3.35)	37.11 (3.40)	0.010*	32.15 (4.16)	35.59 (3.21)	0.014*
	Change	-0.87 (1.43) p = 0.028*	-0.61 (1.08) p = 0.040*	0.56	-0.94 (1.87) p = 0.063	-0.47 (0.97) p = 0.068	0.38
	Percent change	-2.29 (4.39) p = 0.054	-1.50 (2.85) p = 0.052	0.55	-2.62 (5.98) p = 0.10	-1.28 (2.62) p = 0.070	0.42

Data are shown as mean (standard deviation), with volume in ml, unless otherwise indicated; MS = multiple sclerosis; NC = normal controls; total DGM = cerebral subcortical deep gray matter = thalamus + caudate + putamen + globus pallidus; FSL-FIRST = FMRIB’s integrated registration & segmentation tool, v. 5.0

*p<0.05

### Clinical change on-study

Within the MS group, there was no significant change in EDSS scores between baseline [mean±SD (median, range) 1.3±1.0 (1.25, 0–3.5)] and 5 years [1.3±1.0 (1.5, 0–3.5)] (p = 0.47). The T25FW increased from baseline [4.4±0.6 (3.5–5) seconds] to follow-up [4.9±0.8 (4.0–6.1) seconds] over 5 years; this worsening trended to statistical significance (p = 0.054).

### Scan-rescan reliability and effect of scanner upgrade

Table 4 shows scan-rescan variability, both without and with an intervening scanner upgrade. For each data column in the table, 8/11 (72.7%) of the coefficient of variations were less than 1%, which indicates high reliability. This included both the scan-rescan results without an intervening upgrade and a pre- vs. post-upgrade scan-rescan experiment.

**Table 4:** Volumetric measures from MRI: Scan-rescan reliability.

	MS (n = 4), NC (n = 7)	MS (n = 2), NC (n = 1)
Pipeline	Scan-rescan mean COV (%) without an intervening scanner upgrade	Pre-upgrade vs. post-upgrade mean COV (%)
SPM (BPF)	0.49	2.37
FSL-FIRST		
Thalamus	0.38	0.76
Caudate	0.07	0.04
Putamen	1.00	0.07
Globus pallidus	1.14	0.54
Total DGM	0.36	0.28
FreeSurfer		
Thalamus	0.23	2.87
Caudate	0.88	0.40
Putamen	0.64	0.14
Globus pallidus	1.13	3.11
Total DGM	0.57	0.86

Variability is expressed as the coefficient of variation (COV) = (standard deviation/mean)x100%; MS = multiple sclerosis; NC = normal controls; BPF = brain parenchymal fraction; total DGM = cerebral subcortical deep gray matter = thalamus + caudate + putamen + globus pallidus; n = number of subjects receiving scan-rescan pairs; SPM = statistical parametric mapping, v. 12; FSL-FIRST = FMRIB’s integrated registration & segmentation tool, v. 5.0

## Discussion

In this 5-year “real world” 3T MRI study of mildly disabled treated patients with RRMS, although patients began the study with whole brain atrophy compared to normal controls, there was no significant ongoing whole atrophy on-study, compared to healthy controls. However, the DGM (i.e. the caudate nucleus) showed significant atrophy in the MS group over 5 years compared to the rate of volume loss in normal controls. The detectability of caudate atrophy was dependent on the type of automated MRI segmentation pipeline employed. In general, the volumetric DGM measures were not interchangeable between the two automated regional volume segmentation pipelines.

Our findings underscore the increased sensitivity gained when assessing DGM vs. global (whole brain) atrophy in monitoring MS. In considering our observation that DGM atrophy was more sensitive to change than whole brain atrophy in MS, a growing body of evidence supports these results. It is well known that the GM is affected early and selectively in the disease course of MS [7, 11, 33–38]. In addition, several studies indicate that the progression of GM pathology is not necessarily dependent on the extent of WM lesions, supporting the concept that GM degeneration proceeds in a manner largely independent of WM inflammation. Our study is supported by results indicating that subcortical DGM, but not cortical atrophy develops early in people with RRMS [11, 39]. The relevance of DGM has been shown in several studies, such as the observation that selective regional GM, but not global atrophy is an early risk factor for disease progression [40–43]. These results have implications for planning of clinical trials aiming to demonstrate neuroprotective effects of putative MS therapies, for which DGM atrophy may have a role in evaluating treatment outcome [6, 17, 44].

For the measurement of regional DGM volumes, our study showed different results depending on the segmentation pipeline employed. Both software analysis pipelines were able to demonstrate significant regional DGM atrophy in both groups. However, the topography of atrophy detection differed between pipelines. Furthermore, only one of the pipelines showed a higher rate of atrophy in the MS group as compared to the NC group. In a recent study, MSmetrix, FreeSurfer, FSL and SPM were compared for differences in brain volumetric segmentation and showed differences among pipelines [27]. Another recent study [26] showed that GM volumes obtained from FreeSurfer, FSL and SPM were divergent, especially for cortical regions, and that these results affected the strength of correlations between regional GM volumes and clinical/cognitive variables. Recent research also showed a similar discordance of results in DGM volume measurements comparing FSL-FIRST and FreeSurfer pipelines [45]. These studies are consistent with our findings.

The reasons for such differences between software pipeline is difficult to pinpoint since they employed fundamentally different methods. FSL-FIRST [46] registers the individual scan to a standard space brain (derived from the MNI-152 atlas) and models the outer surface of each DGM structure as a mesh, and, finally, assigns each voxel in the image the appropriate label to indicate the structure. This takes into account local variations in structure surface shape, as well as the presence of neighboring structures. On the other hand, FreeSurfer [47, 48] performs volume analysis for the DGM structures in native space. Differences between these software packages could arise from the segmentation itself, the atlas used, or the smoothing kernel used in voxelwise analyses. The lack of a generally accepted gold-standard for regional atrophy measurements including all brain structures, limits the assessment of false negative or false positive voxels. The major implication of these results is the need to maintain consistency in the type of analysis pipeline employed to avoid potentially severe biases that may occur when pooling data from different methods [49]. In conclusion, DGM volumes obtained from different image analysis methods can be very different.

There are several limitations of our study to be considered. Care should be exercised in interpreting these results because of the relatively small sample size. Second, our study was only limited to mildly affected individuals with RRMS. The results may not necessarily apply to other stages of MS, such as more active or progressive patients, including those with primary or secondary progressive forms of the disease. Also, due to limited power, we could not properly evaluate the clinical relevance of our results such as how these volumetric biases would affect clinical-MRI correlations or the assessment of therapeutic response.

## Supporting information

S1 File Spreadsheet for segmentation data. The spreadsheet includes all study subjects’ data including demographic and clinical characteristics, as well as segmentation data calculated from all four pipelines (SPM12, SIENA, FSL-FIRST and FreeSurfer).
(XLSX)
